# Revisiting the Role of Exosomes in Colorectal Cancer: *Where Are We Now?*

**DOI:** 10.3389/fonc.2019.00521

**Published:** 2019-06-19

**Authors:** Francesco Mannavola, Tina Salerno, Anna Passarelli, Marco Tucci, Valeria Internò, Francesco Silvestris

**Affiliations:** Department of Biomedical Sciences and Human Oncology, University of Bari Aldo Moro, Bari, Italy

**Keywords:** extracellular vesicles, exosomes, colorectal cancer, liquid biopsy, engineered nanovesicles

## Abstract

Exosomes (Exos) are nano-sized extracellular vesicles constitutively released by both prokaryotic and eukaryotic cells. Their role as inter-cellular messengers involved in both physiological and pathological processes has overwhelmingly come to light in the last decade, and their contribution to cancerogenesis and tumor metastasis is under intensive investigation. Here we review the most recent information concerning Exos in colorectal cancer (CRC) and focus on their effects on tumor microenvironment and the immune system, as well as unravel their role in the formation of the pre-metastatic niche and in drug resistance. Such a recent knowledge on Exos depicts their potential translations into the clinical arena, either as an alternative tool of “liquid biopsy” or novel therapeutic approaches for CRC. However, due to the limited data available from clinical trials, they need further validations before addressing their putative application in oncology.

## Introduction

The constitutive activation of the RAS-RAF mitogen-activated protein kinase (MAPK)-driven intracellular signaling, together with the angiogenic switch, have been widely described in the malignant transformation of CRC (1). However, the tumor microenvironment plays a pivotal role for the tumor progression including the crosstalk of tumor cells with the surrounding normal counterparts, as mesenchymal and immune cells, and promotes tumor growth and metastasis, while impairing the immune system activity (2). A complex network of connections is, indeed, needed to assure the adequate inter-cellular communication within the tumor milieu, mainly depending on direct cell-to-cell contacts or on the secretion of different paracrine signals, such as growth factors and inflammatory cytokines (3). Recent data also reveal an alternative mechanism for intercellular signaling based on the release of different kinds of extracellular vesicles (EVs), including exosomes (Exos), microvesicles, and large-oncosomes (4).

In the past few years, Exos have been widely investigated since they were discovered in many physiological and pathological processes, and accumulating evidence support their role also in CRC (5). These small (50–130 nm) EVs are detectable in most body fluids, such as plasma, urine, saliva, or ascites (6). Unlike other EVs, which directly bud-off from the cell membrane, Exos are end-products of recycling endosomal pathways since they originate from inward budding of the plasma membrane, with the subsequent formation of multivesicular bodies (MVBs). These complex structures encapsulating early Exos are actively loaded with a number of molecules including proteins, coding- and non-coding RNAs as well as DNA fragments (7, 8). This machinery produces mature Exos that are released in the extra-cellular space by fusion of MVBs with the cell membrane. Thus, vesicles are spread in the blood stream (9).

Although Exos were originally considered as cellular waste products, it is now accepted that they have a key role in intercellular communication, depending on the delivery of their cargos from donor to distant cells (10, 11). In this context, Exos from CRC cells were found to promote proliferation, migration, and invasion of cancer cells, as well as to affect both angiogenesis and immune-system activity. Also, Exos have recently emerged as possible players in gaining resistance to both cytotoxic and targeted agents that are commonly used to treat patients with metastatic CRC (mCRC). However, additional efforts are required to translate Exos in clinical practice, and their therapeutic potential is currently under investigation.

Here, we review recent studies on the biological functions as well as both diagnostic and therapeutic implications of Exos in CRC.

## Exosomes and Tumor Progression

Major events driving the cancerogenic process of CRC include the acquisition of an invasive phenotype and the orchestration of a newly vascular network surrounding the tumor bed. Exosomes, indeed, take part in all of these processes by promoting both proliferation and invasiveness of cancer cells, as well as by supporting the angiogenic switch and the remodeling of the extracellular matrix (ECM).

### Cell Proliferation

Apart from acquired mutations affecting driver genes that regulate the cell cycle and apoptosis, such as *RAS* or *p53* (12), the signals promoting cancer cell proliferation also depend, at least in part, on an epigenetic regulation of genes exerting either suppressive or oncogenic functions (13). These genes may be negatively modulated by microRNAs (miRNAs), namely short (19–21 nucleotides) non-coding RNAs, and those affecting proto-oncogenes are generally referred to as onco-suppressive miRNAs since their over-expression was found to protect the normal cells from cancer transformation by dampening the proliferative signals (14). In this context, a constitutive removal of onco-suppressive miRNAs is required by tumor cells to increase their proliferative extent, while it has also been demonstrated that CRC cells may decrease the cytoplasmic amounts of such miRNAs through a direct exosomal garbage mechanism. These properties have been recently discovered by Teng who demonstrated the selective sorting of suppressive miR-193a by CRC cells into their nanovesicles and the consequent discharge in the extracellular space (15). The authors, indeed, found a direct anti-proliferative effect of miR-193a by targeting Caprin1, namely a positive regulator of the cell cycle.

Similarly, CRC cells may release Exos to get rid of both transmembrane (16) and soluble proteins (17), which are able to promote the proliferation of recipient cells in a paracrine manner. In this context, the release of CD133^+^ Exos by poorly differentiated CRC cells was found to increase the phosphorylation of Src and ERK in surrounding cells, with consequent activation of MAPK intracellular signaling and promotion of tumor growth (16).

### Migration and Invasiveness

Exosomes from *KRAS-mutated* colon tumors were found to enhance the *in vitro* invasiveness of recipient cells. These Exos can transfer many tumor-promoting proteins (e.g., mutant-KRAS, EGFR, and integrins) to *KRAS-wild type* cells, thus enhancing their three-dimensional growth and migratory properties (18). Also, tumor-derived Exos stimulate CRC cells to activate the epithelial-to-mesenchymal transition (EMT) machinery and promote their invasive behavior by loss of epithelial characteristics in favor of mesenchymal-like phenotype (19). The delivery of miR-210 *via* Exos, indeed, was recently identified as one of the possible mechanisms promoting EMT in colon cancer, since the over-expression of this miRNA was associated with reduced cell-to-cell interactions as well as with increased cell motility and invasiveness (20). Therefore, it is conceivable that cancer cells with aggressive phenotype can promote EMT in neighboring cells through an Exo-mediated transfer of pro-tumorigenic factors, thus inducing the onset of invasive and pro-metastatic features to non-aggressive cancer cells.

### Angiogenic Switch

Angiogenesis is a complex process driven by several factors that is mostly deregulated in cancer. The increased secretion of vascular endothelial growth factor (VEGF) by cancer cells as an adaptation to hypoxic tumor microenvironment has been proposed as a main mechanism involved in the angiogenic switch of CRC (21). However, during the early phases of tumor progression, a number of different stress conditions including hypoxia, stimulate the colon cancer cells to increase the Exos release, probably by triggering some innate survival mechanisms that are still unclear (22). These Exos engulfing the tumor milieu are internalized by endothelial cells and promote their proliferation and migration in a VEGF-independent manner, which is influenced by the vesicles' cargo. The content of tumor-derived Exos changes when colon cancer cells are cultured under hypoxic conditions and their enrichment with several cell cycle-related mRNAs and Wnt family proteins, has been correlated with the proliferative effect of these vesicles on endothelial cells through the activation of β-catenin intracellular signaling (23, 24).

### Cross-Talk With Stromal Cells

The tumor microenvironment is a heterogeneous and dynamic network including both cancer and stromal cells, as cancer-associated fibroblasts (CAFs) (25). Several tumorigenic signals are derived from tumor cells and conveyed to stromal cells through Exos. Since the early phases of CRC progression, in fact, stromal accessory cells are prompted by tumor-derived Exos to display a highly pro-proliferative and pro-angiogenic phenotype (26). Additional features of CAF activation by Exos include the metabolic reprogramming as well as the enhanced secretion of ECM-remodeling proteins, thus facilitating tumor growth and metastasis.

Recently, CAFs have been investigated as an active source of Exos interplaying with cancer cells. In this context, Bhome et al. have shown that CAF-derived Exos can interact with CRC cells promoting *in vitro* proliferation and chemoresistance in relation to the specific cargo of miRNA (27). Indeed, using an orthotopic murine model, these authors demonstrated that Exo-mediated transfer of miR-21 from CAFs to cancer cells increases their metastatic potential, accordingly to previous reports, attributing to this miRNA a defined inhibitory activity toward different onco-suppressor genes, such as *PTEN* and *PDCD4* (28). These data also endorse previous retrospective observations that high miR-21 expression measured on the stromal component of resected colorectal tumors correlates with a short relapse free survival (27).

### Pre-metastatic Niche Formation

Finally, tumor-derived Exos drive the dissemination of cancer cells from primary site toward distant organs by preparing a niche suitable for their homing and outgrowth. This complex sequence of events, namely pre-metastatic niche formation, depends on a variety of pro-metastatic signals propagated by tumor cells, including cytokines, growth factors, and Exos, that start their remodeling of the future metastatic bed even before the arrival of cancer cells (29). In this context, Exos deliver a cargo of active molecules that promote the secretion of angiogenic factors, matrix metalloproteinases (MMPs), and immune-suppressive cytokines (30). In an elegant study, Hoshino et al. demonstrated that tumor-derived Exos also influences the organotropism of metastatic cancer cells by driving their destination toward predicted sites (31). Hence, exosomes disseminating throughout the bloodstream are captured by specific organs depending on the exosomal integrin repertoire, which drives their binding with resident target cells. Therefore, it is conceivable that Exos can start the formation of the pre-metastatic niche only in those sites permissive for their anchorage and fusion.

Concerning CRC, Takano et al. showed that tumor-derived Exos are able to initiate the formation of the hepatic pre-metastatic niche *in vivo* and proposed the exosomal transfer of miR-203 as a mechanism putatively implicated in this process that promotes the differentiation of monocytes into M2-tumor associated macrophages (TAMs) (32). This model suggests that CRC-derived Exos may favor liver metastasis by acting as intercellular messengers between tumor and immune cells through the recruitment of TAMs at the future metastatic niche, although further investigations are needed to confirm this hypothesis. In line with this suggestion, a parallel study revealed that a possible effect of Exos released by CRC cells may be the activation of the SDF1-mediated chemotaxis of stromal cells toward the hepatic niche (33). This supports the interpretation that Exos promote liver metastasis from colorectal tumors by recruiting CXCR4-expressing cells, even including immune cells, endothelial cells, fibroblasts, bone marrow-derived cells and stem cells, to develop a suitable pro-metastatic microenvironment.

## Exosomes and Immune System Activity

**Figure 1:**
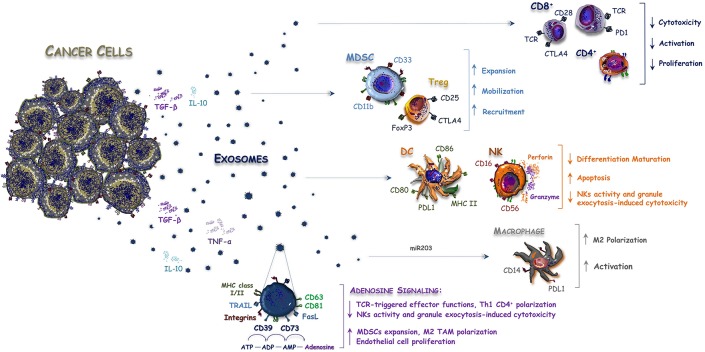
Exosomes released by CRC cells balance immune system activity. Anti-cancer immune response is modulated within the tumor microenvironment by interleukins and Exos released by CRC cells. Exosomes contain both inhibitory and stimulatory molecules that alter the immune system balance by favoring the expansion, mobilization, and recruitment of Tregs and MDSCs or by blocking the activity of CD8+ T-cells, DC, and NK cells. On the other hand, miR-203 bearing Exos are released by CRC cells and internalized by monocytes to promote polarization and activation of M2-macrophages, while CD39/CD79+ Exos increase the amount of immunosuppressive adenosine signaling within the tumor microenvironment, thus further inhibiting the anti-tumor immune response.

A critical step for the tumor development is the establishment of an immuno-suppressive microenvironment, mainly induced by chronic hypoxia, and inflammation (34, 35). Tumors originating from the intestinal epithelium escape the recognition by the immune system, similarly to other immune-privileged sites that employ several mechanisms as the expression of pro-apoptotic molecules of the tumor necrosis factor (TNF) family, such as Fas ligand (FasL), and TNF-related apoptosis-inducing ligand (TRAIL) (36, 37). Tumor-derived Exos are also involved in the spreading of immuno-suppressive signals and contribute to the impairment of an effective immune response within the tumor microenvironment by affecting proliferation, maturation and antitumor activity of immune cells (38, 39). According to other tumor histotypes (40), Exos from CRC cells deliver death signals to anti-tumor immune cells, thus bypassing the direct cell-to-cell interaction (Figure 1). In this regard, Huber et al. have shown for the first time that CRC-derived vesicles deliver different immuno-suppressive signals, such as FasL and TRAIL, which induce the apoptosis of CD8^+^ T lymphocytes (36). Noteworthy, FasL- and TRAIL-bearing vesicles have been isolated from plasma of CRC patients to support their potential role in modulating the immune system activity and suggesting their use as prognostic biomarker. In addition, Exos contribute to the release and the production of extracellular adenosine, that is a potent negative regulator affecting the T-cell functions (41). In this regard, it has been demonstrated that Exos derived from different cancer cells including bladder, prostate and colorectal tumors, express both CD39 and CD73 ecto-nucleotidases (42) that are involved in the adenosine triphosphate (ATP)-de-phosphorylation to adenosine (43).

On the other hand, an indirect role of Exos has also been described in the expansion of negative regulators of the immune system, such as myeloid-derived suppressor cells (MDSCs), regulatory T-cells (Tregs), and monocytes. These cells favor at all the tumor escape from the immune surveillance (40, 44). In this context, miR-203 bearing Exos are released by CRC cells and internalized by monocytes, thus promoting the expression of M2 markers (32). This suggests a potential involvement of exosomal miRNAs in the differentiation of monocytes into M2- tumor-associated macrophages (TAMs), whose immunosuppressive role in CRC has been described (45). Accordingly, a high expression of miR-203 detected in serum Exos from CRC patients is an independent poor prognostic factor, correlated with increased metastatic potential and short survival (32). Also, the immune-modulating effect of intestinal microbial EVs was recently reported to cooperate in M2-macrophage polarization (46, 47).

Although these studies support that Exos contribute to the immune escape process of CRC, their definite role in the anti-tumor immune response is still debated and further hypotheses suggest, on the contrary, a possible promotion of both adaptive and innate immunity through various mechanisms. Gastpar et al. also demonstrated that CRC cells release Exos carrying a membrane-bound complex of the heat shock protein 70 (Hsp70), which can stimulate the migration and cytolytic activity of natural killer (NK) cells (48). Noteworthy, a previous work also documented an extracellular secretion of the Hsp70 exerting regulatory effects on human monocytes, thus suggesting a dual role as chaperone and cytokine, both exerting pro-inflammatory effects (49).

## Exosomes and Resistance to Anticancer Agents

**Figure 2:**
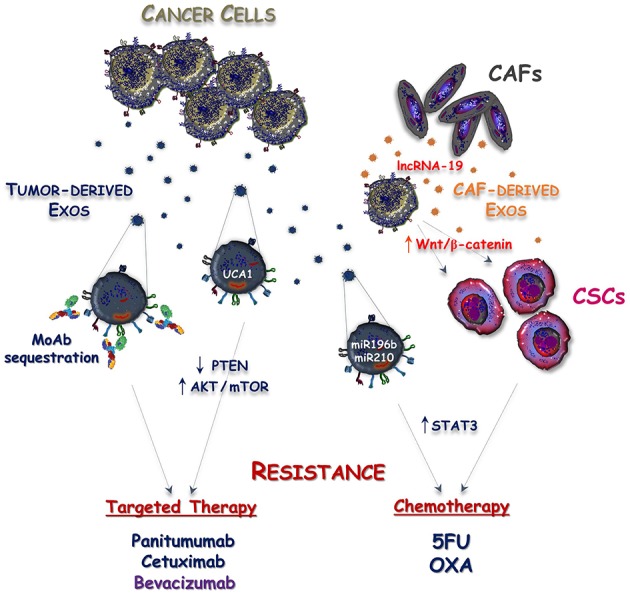
Mechanisms responsible for Exosome-mediated drug resistance in CRC. Exosomes released by either tumor cells (Tumor-derived Exos) or cancer associated fibroblasts (CAF-derived Exos) cooperate to induce resistance of CRC against targeted and cytotoxic drugs. This process is mediated by exosomal delivery of non-coding RNAs into cancer cells leading to the stimulation of mTOR and STAT3 intracellular signaling, as well as to the acquisition of cancer stemness features. Additionally, sequestration of anti-EGFR (panitumumab and cetuximab) and anti-VEGF (bevacizumab) monoclonal antibodies (MoAbs) by Tumor-derived Exos is another possible mechanism reducing the bioavailability of these drugs and defective anti-cancer activity.

Despite tumor genomics and immunotherapy have overwhelmingly progressed during the last two decades, chemotherapy remains a backbone of mCRC treatment. A better understanding of the mechanisms regulating both primary and acquired drug resistance is thus an urgent need to improve the survival of these patients. To this regard, Exos released by either cancer or stromal cells may have a pivotal role in these processes and may contribute to tumor resistance against either cytotoxic or targeted agents (Figure 2).

### Chemotherapy

Apart from classical mechanisms associated with resistance to 5-fluorouracil (5-FU) and oxaliplatin (OXA), including the alteration of cellular drug influx/efflux, enhancement of drug inactivation and single nucleotide polymorphisms (SNPs) of fluoropyrimidine or platinum targets (50), the acquisition of cancer stemness has been proposed as a possible way to induce chemoresistance in CRC (51). Cancer stem cells (CSCs) are endowed with indefinite self-renewal activity and maintain the ability to generate both tumorigenic and non-tumorigenic cells. Their innate drug resistance depends on the increased expression of membrane multidrug transporters, such as ABCG2 and ABCB5, as well as on their intrinsic low proliferative rate, resulting in efficient drug discharge, and reduced sensitivity to chemotherapeutics (52). The over-expression of miR-196b-5p has also been identified as an additional hallmark of cancer stemness and resistance to 5-FU *via* targeting SOCS1 and SOCS3, namely two negative regulators of STAT3 signaling pathway (53). Interestingly, the miR-196b-5p was found highly enriched in serum Exos of patients with CRC compared to healthy subjects, suggesting an active participation of these vesicles in maintenance of both stemness and chemoresistance of CRC cells. Despite no progress being reached by this hypothesis, additional studies revealed that CAF-derived Exos deliver lncRNA-19 to colon cancer cells, thus priming their stem cell properties and drug resistance by activating the Wnt/β-catenin pathway (54, 55). Furthermore, the activation of ERK/AKT pathway by CAF-derived Exos was recently reported to exert a protective effect on CRC cells in the presence of OXA (27).

### Targeted Agents

Panitumumab and cetuximab are two monoclonal antibodies (mAbs) targeting the extracellular domain of the epidermal growth factor receptor (EGFR). The binding of these mAbs to EGFR prevents the activation of MAPK pathway and their use in combination with chemotherapy produces a significant survival benefit in patients with *RAS*-*wild type* mCRC (56). Several gene alterations, such as *BRAF* and *PIK3CA* mutations, as well as *MET* and *HER2* amplification, were robustly correlated with primary resistance to cetuximab, while the activation of parallel RAS-independent pathways, including the PI3K/AKT/mTOR cascade, drives the acquired to anti-EGFR mAbs (57–59). In this regard, several mechanisms are involved with paradoxical activation of mTOR signaling (60) and a recent study suggested a possible effect of Exos from cetuximab-resistant CRC cells to restrict the expression of PTEN, a negative regulator of PI3K (61). Although the exact contribution of Exos in the modulation of PTEN was not explored in this work, Yang et al. found that Exos released by cetuximab-resistant CRC cells expressed high levels of UCA1-lncRNA and demonstrated that exosomal delivery of UCA1 to cetuximab-sensitive cells induced resistance to anti-EGFR agents (62). Noteworthy, although in a different cancer model, UCA1 up-regulation was described to induce acquired resistance to EGFR tyrosine kinase inhibitors (TKIs) in *EGFR-mutant* NSCLC, and this was correlated to the activation of the AKT/mTOR pathway (63).

Novel mechanisms of resistance mediated by CRC cell-derived Exos are now emerging since loading of EGFR on exosomal membrane has been recently revealed (64). Thus, EGFR-containing Exos may bind the circulating anti-EGFR mAbs, acting as decoy receptors to reduce drug bioavailability. Although this hypothesis is currently under investigation, a similar mechanism has already been described to drive acquired resistance to bevacizumab (65), namely a mAb directed toward the vascular endothelial growth factor-A (VEGF-A) that is commonly used in association with chemotherapy, also in patients affected by glioblastoma (GMB) as well as metastatic breast and lung cancer. In this context, Simon et al. showed that bevacizumab can be detected at the surface of GMB-derived EVs and further demonstrated that this mechanism is used by cancer cells for the antibody neutralization (65).

## Clinical applications of Exosomes

Extracellular vesicles are normally released by all cells, thus purifying tumor-derived Exos from body fluids (e.g., plasma, urine, and saliva) is an attractive tool for both diagnostic and prognostic purposes. Other applications of Exos may include the selective drug delivering into tumor cells and the stimulation of immunological response against cancer cells.

### Circulating Biomarkers

Since tumor-derived RNAs and DNAs packaged within the exosomal phospholipidic bilayer are protected from degradation by serum ribonucleases and DNases (66), their analyses provided additional diagnostic and prognostic information for cancer patients and are currently termed as “liquid biopsy” (67). In this context, seven miRNAs (let-7a, miR-1229, miR-1246, miR-150, miR-21, miR-223, and miR-23a) were found significantly over-expressed in serum Exos from patients with colorectal tumors at various stages, while being undetectable in healthy subjects (68). Thus, measuring serum levels of these exosomal miRNAs may be considered in the average-risk population as a non-invasive screening test for CRC diagnosis. Another study demonstrated that miR-125a-3p and miR-320c were significantly up-regulated in plasma-derived Exos from patients with localized colon tumors, while the combination of exosomal miR-125a-3p and CEA levels significantly increased the diagnostic power of early CRC (69).

Other Exo-miRs (e.g., miR-17-92a, miR-92, miR-638, and miR-19a) were classified as negative prognostic factors of CRC. Elevated serum levels of these Exo-miRs were indeed variably correlated with lymphatic/vascular infiltration or short relapse-free survival (RFS), thus identifying novel prognostic biomarkers to recognize those patients at high risk of recurrence after tumor resection early (70–72).

Thus, both isolation and characterization of tumor-derived Exos from body fluids may be used to predict the responsiveness to targeted agents. To this purpose, Hao et al. demonstrated a high concordance of KRAS/BRAF mutational status between primary tumor and serum Exos in patients with CRC, thus representing a possible replacement of tumor biopsy when rapid and non-invasive genotyping is required (73). Moreover, as already described, serum levels of exosomal UCA1-lncRNAs may be measured for the identification of patients harboring *RAS*-*wild type* mCRC with primary resistance to anti-EGFR mAbs (62).

Finally, another important issue of circulating Exos includes the possibility to isolate and analyze EVs originating from immune cells. In this context, the phenotypic profile of immune cell-derived Exos is a *bona fide* representation of the status of immune system activation and may reflect the propensity to respond to immunotherapy, as recently reported in melanoma (74). Although immunotherapy in mCRC has demonstrated efficacy only in a subset of patients with high microsatellite instability (MSI-H) tumors, immune cell-derived Exos may become of great interest in the near future, since novel immunotherapy strategies aimed at converting immune-desert into immune-inflamed tumors are currently under investigation (NCT01633970; NCT01988896; NCT02650713; NCT03832621).

A current limitation of Exos for diagnostic purpose, however, is the lack of standardized and universally accepted methods for both nanovesicle isolation and downstream analyses. Particularly, despite several commercially kits being available for rapid and easy purification of Exos, a suitable yield still requires the ultracentrifugation of large volumes of biological fluids. This greatly limits the applicability of Exos as a high-throughput diagnostic tool, while the specificity and sensitivity of this approach result was reduced by the interference of body fluid nanovesicles with plasma proteins and immunoglobulins, as well as circulating-free nucleic acids (75).

### Therapeutic Applications

One of the major unmet issues in CRC is targeting mutated forms of RAS kinases since these are considered largely undruggable (76). Thus, alternative strategies including non-coding RNAs have been investigated in other tumor models, such as pancreatic (77) and lung cancer (78, 79), for directly inhibiting the downstream transduction of the *RAS*-mutated gene. Small interfering-RNAs (siRNAs) for specific *KRAS* point mutations have shown attractive anti-tumor activity in non-small cell lung cancer (NSCLC) and may be directly translated in CRC models. However, the high polarity and molecular size of these molecules reduce their capacity to be stably transfected into target cells and limit their translation in clinical trials (80). Thus, innovative systems for RNA-delivering are now under investigation, including nanoparticles, liposomes, and engineered Exos. To this purpose, Exos from normal fibroblast-like mesenchymal cells have been developed to carry specific siRNAs or short hairpin-RNAs (sh-RNAs) to *KRA*S^*G*12*D*^ pancreatic cancer cells and promising anti-tumor activity in multiple mouse models has been demonstrated (81). Additionally, the composition of the phospholipidic bilayer of Exos, compared to other lipo-particles, protects them from the phagocytosis by the reticuloendothelial system contributing to their diminished blood clearance, and hence rendering Exos a suitable vector for efficient siRNA delivery to cancer cells (82, 83).

Exosomes from dendritic cells (Dex) have also been extensively investigated for their contribution to induce antigen-specific T-cell responses and tumor growth regression (84–86) and early phase clinical trials have been designed to investigate their potential use as cell free anti-tumor vaccines (87–89). However, based on the limited efficacy of immunotherapy in the majority of colorectal tumors, to date Dex have not yet been investigated in this context. In 2008, a pivotal phase I trial used ascites-derived exosomes (Aex) in combination with GM-CSF to treat 40 advanced CRC patients (90). Exosomes purified from malignant ascites were found to be enriched in MHC-I and MHC-II, as well as in immunogenic carcinoembryonic antigen (CEA). Despite a good safety profile was ascribed to Aex, no detectable therapeutic responses were revealed except for stable disease in a couple of patients.

## Conclusions

Exosomes exert a wide range of biological functions, primarily via delivering signaling molecules that regulate diverse cellular processes. Because they also contribute to CRC development and metastasis, their detection in a variety of biological fluids represents a very easy and reproducible strategy to achieve pathogenic information and to identify specific biomarkers of diagnostic and prognostic relevance. Moreover, besides the efficacy of combining chemotherapy with anti-EGFR targeted therapy in *RAS wild type* tumors, modest progress has been achieved against *RAS-mutated* mCRC and intelligent nanoparticle systems for gene therapy approach are currently under investigation.

However, although pre-clinical data appear very promising, validation from large clinical trials are needed to support the applicability of Exos as tumor biomarkers for monitoring cancer progression and driving treatment decisions. These findings are necessary to improve our understanding of the role of Exos in cancer progression and to translate their use in clinical practice.

## Author Contributions

All authors listed have made a substantial, direct and intellectual contribution to the work, and approved it for publication.

### Conflict of Interest Statement

The authors declare that the research was conducted in the absence of any commercial or financial relationships that could be construed as a potential conflict of interest.
